# Early Effects of Communities That Care on the Adoption and Implementation Fidelity of Evidence-Based Prevention Programs in Communities: Results from a Quasi-experimental Study

**DOI:** 10.1007/s11121-025-01823-w

**Published:** 2025-07-01

**Authors:** L. Decker, I. von Holt, S. Ünlü, U. Walter, D. Röding

**Affiliations:** Hannover Medical School, Institute for Epidemiology, Social Medicine and Health System Research, Carl-Neuberg-Str. 1, 30625 Hannover, Germany

**Keywords:** Community-based health promotion, System change, Evidence-based prevention and health promotion, Quasi-experimental study, Implementation fidelity

## Abstract

To date, few evidence-based prevention programs (EBP) have been implemented in Germany, despite their growing availability. Some prevention researchers have stated that prevention support systems such as Communities That Care (CTC) could contribute to the wider spread of EBP in Germany and improve the fidelity of EBP implementation. Our study aims to investigate whether CTC leads to greater adoption of EBP in German communities. A quasi-experimental study was conducted with 22 intervention communities (IC) and 22 comparison communities (CC). Data collection occurred at two time points (T0: 2021/2022 and T1: 2023/2024), capturing information on which EBP were implemented in these communities during the prior school year, the number of people reached, and the quality of EBP implementation. Data from 17 IC and 12 CC were included in the final analyses. At T0, there were no significant differences between IC and CC in the number of EBP implemented or the number of people reached. In the IC, the average number of EBP implemented was 3.57 per 10,000 residents at T0, which increased significantly to 8.57 at T1 (*p* = .004). In the CC, the average number rose from 1.88 at T0 to 3.41 at T1, though this change was not statistically significant (*p* = .089). Regarding reach, an average of 140 people per 10,000 residents were reached with EBP at T0 in IC, increasing to 407 by T1, while in CC, the average increased from 77 at T0 to 300 at T1. Neither group showed a statistically significant increase in reach. Implementation fidelity remained consistently high in both groups at both time points. The results suggest that CTC may encourage communities to implement more EBP. However, the findings may be influenced by confounding factors and the relatively short observation period. Further research with an extended follow-up is therefore recommended.

**Supplementary Information:**

The online version contains supplementary material available at 10.1007/s11121-025-01823-w.

## Introduction

A key goal of prevention science is to provide a wide range of proven, effective prevention programs (Glasgow et al., 2003, 2012; Rohrbach et al., 2006). In the past, Germany has lagged in this area, and the use of evidence-based prevention programs (EBP) has been uncommon (Frantz et al., 2015; Ghanem et al., 2017; Karing & Beelmann, 2019; Karing et al., 2015). However, there are now calls to establish and expand registries of EBP (Rossmann et al. 2021; Trojan et al. 2021; Beelmann & Karing, 2014). Additionally, little research has been conducted on the fidelity and reach of EBP in Germany (Karing et al., 2015; Piontek et al., 2013; Schloemer et al., 2021). It is well known that insufficient fidelity and inadequate adaptation to the local context can undermine the effectiveness of EBP (Carroll, 2020; Durlak & DuPre, 2008; Fiedler et al., 2020; Jantzer et al., 2023).

Some prevention researchers have stated that prevention support systems (Chinman et al., 2019) such as Communities That Care (CTC) could contribute to the wider spread of EBP in Germany and improve the fidelity of EBP implementation (Beelmann & Karing, 2014; Frantz et al., 2015). CTC involves the formation of diverse and broad-based coalitions that receive training and technical assistance on how to identify elevated risk factors and depressed protective factors faced by community youth (Hawkins & Catalano, 2002). These coalitions then target these needs using prevention measures and programs that have previously been tested and proven effective, and monitor the implementation quality of selected measures and programs (Hawkins & Catalano, 2002). The CTC approach, developed in the late 1980s, was first piloted in Germany in a pilot project from 2009 to 2012 (Groeger-Roth, 2010, 2012). Since then, the approach has been spreading in Germany and is currently implemented in approximately 50 communities (Walter et al., 2023). In addition, a National CTC Transfer Centre has been established to provide CTC training and technical assistance. Detailed information can be found on the official website of the German version of CTC (www.ctc-info.de).

**Fig. 1:**
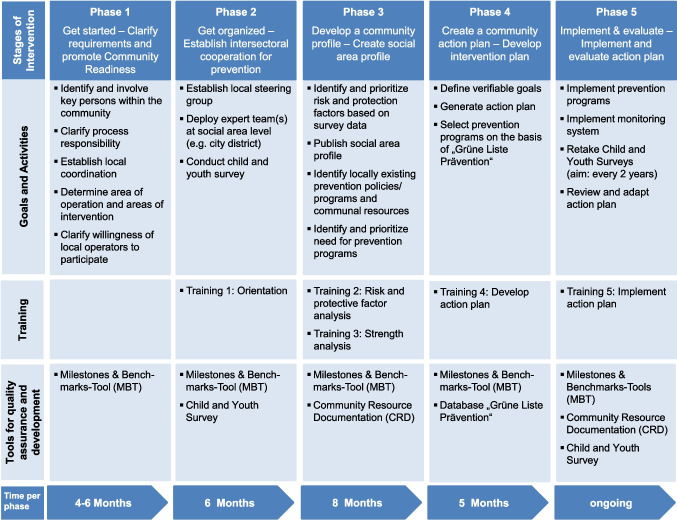
Overview of the core components of CTC

The implementation of CTC takes place in five phases, which together cover a period of at least 24 months (Walter et al., 2023). The objectives, activities, training, and instruments in each phase are depicted in Fig. 1. Typically, in phase 4, CTC communities select EBP from the ‘Grüne Liste Prävention’ (http://www.gruene-liste-praevention.de) (Brender et al., 2024) based on their specific needs in order to implement them locally. It can therefore be assumed that CTC contributes to the dissemination of EBP, thereby reaching more people with these programs in CTC communities. As the trained CTC community coalitions are also supposed to monitor the fidelity of implementation, it can be assumed that CTC communities implement EBP with a higher degree of fidelity. The Community Youth Development Study (CYDS), a community-randomised trial to evaluate CTC that was conducted in the USA from 2003 to 2007, showed that more EBP were implemented after the introduction of CTC than in the control communities and that more people were reached with EBP (Fagan et al., 2011, 2012). However, there were no differences in the fidelity of EBP implementation between CTC and control communities. According to the CTC theory of change, this increase in the number of EBP implemented in communities leads to better prevention outcomes for adolescents in CTC communities.

In our study, we investigate for the first time for Germany whether introducing CTC leads communities to implement more EBP and reach more people with EBP. Additionally, we examine whether the implementation fidelity of EBP improves after the introduction of the CTC approach.

## Methods

### Study Design

The following analyses are based on longitudinal data derived from the CTC-EFF study, which is a non-blinded, non-randomised community trial (Röding et al., 2021a, 2021b). The CTC-EFF study was designed as a conceptual replication study of the CYDS. The unit of assignment is the community, and the assignment method is self-selection.

### Setting and Community Sampling

From April 2020 to February 2021, the study recruited 44 small towns, rural communities, or districts of major cities across four German federal states (Bavaria, Lower Saxony, Rhineland-Palatinate, and Baden-Wuerttemberg); hereinafter referred to as communities. In a first step, all German communities that had been in an early phase of introducing CTC were invited as intervention communities (IC). To be included in the study, IC had to have at least one secondary school and willingness to sign a cooperative agreement for study participation with the principal investigator, the Hannover Medical School. As soon as an IC had accepted the invitation, potential comparison communities (CC) in the same federal state were identified using official statistics (e.g. population size, crime rate, and deprivation). In a third step, one CC was recruited for each IC. CC were eligible to participate in the study only if they had at least one secondary school, they were not located in a county that implements CTC, and they were not directly adjacent to a CTC-community. Overall, a sample of 22 matched pairs was recruited. Details of the recruitment and matching process have already been published in detail elsewhere (Röding et al., 2021a, 2021b). However, as the data collection period coincided with the COVID-19 pandemic, which lasted around three years, there was a high drop-out rate among the communities during this time. In the first survey wave (T0), only 17 IC and 12 CC took part in the surveys described below. Only 16 IC and 11 CC took part in the second survey wave (T1). Due to this high drop-out rate, we decided to include all communities for which we have data. Subsequently, the 1:1 matching that was carried out a priori had to be cancelled.

### Survey Sampling and Participants

To identify all evidence-based prevention programs (EBP) in the communities and collect data on these programs, we used the so-called community resource documentation (CRD) procedure developed for this purpose in the CYDS (Fagan et al., 2012; Hawkins et al., 2008). The CRD is based on a snowball sampling process and comprises four questionnaires for four target groups: (1) networks, (2) program agents, (3) school principals, and (4) teachers. The questionnaires were translated into the German language as well as adapted to the German context. They were subjected to a cognitive pre-test before data collection.

**Fig. 2:**
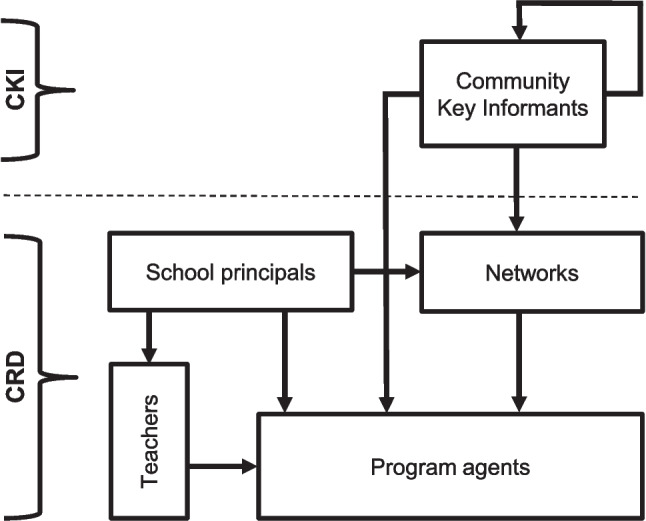
Snowball sampling for identifying program agents. CKI = Community Key Informant Interview; CRD = Community Resource Documentation

Snowball sampling (Fig. 2) began with community key informant interviews (CKI). Community key informants like mayors, school principals, police officers, health officials, and youth work leaders were first identified through internet searches. They were interviewed to determine, among other things, whether they were aware of individuals or organisations implementing prevention programs in their community and, if so, whether they could provide contact information. Furthermore, research was carried out to identify active prevention networks (coalitions) within the communities. These coalitions were then asked about the prevention programs they had implemented in the community and if they could provide contact details of other coalitions or agencies running prevention programs locally. Additionally, school principals were interviewed about school-wide prevention programs implemented at their schools. If principals reported such programs, they were asked to provide the contact details for the person responsible. Principals were also requested to forward an invitation for an online survey to all teachers who may have run a program in their classes. At the end of the teacher survey, respondents were asked if they knew of any other individuals or organisations running prevention programs in their community, and contact details were requested, if available. Every participant signed a written informed consent. Potential interviewees were initially invited to participate via e-mail, which included study information, a data privacy protection document, as well as a formal cover letter requesting participation. If there was no response after two weeks, follow-up contact was made by telephone. If multiple calls went unanswered, reminder e-mails were sent summarising the study content. Data collection for T0 took place from June 2021 to October 2022, and for T1 from October 2023 to March 2024.

### Measurement and Variables

The following outcomes were assessed with the CRD survey: The two primary outcomes are the number of EBP implemented (adoption) and the number of people reached with the EBP (reach). Eight measures of program fidelity were collected as secondary outcomes. Table 1 shows how these outcomes were assessed in the CRD.

**Table 1:** Implementation, reach, and fidelity measures of EBP in the CRD survey

Measure	CRD interview/survey type			
	Agency interview	Coalition interview	Teacher interview	Principal interview
*Adoption*				
Number of implemented EBP	2 items (yes/no; open-ended) With your network’s help, were any of the following evidence-based prevention programs implemented in 2020/21? Which other programs did your network offer for children and teenagers in years 2020/21?	2 items (yes/no; open-ended) With your network’s help, were any of the following evidence-based prevention programs implemented in 2020/21? Which other programs did your network offer for children and teenagers in years 2020/21?	2 items (yes/no; open-ended) Which of the following programs were implemented in years 2020/21? Did you carry out other evidence-based programs in years 2020/21 and if so, how are they called?	3 items (yes/no; open-ended) Which of the following school-based prevention programs were implemented in years 2020/21? Which of the following programs to enhance parenting skills were implemented at your schools in years 2020/21? Did you carry out other prevention activities in years 2020/21 and if so, how are they called?
*Reach*				
Number of persons reached by EBP	1 item (integer) How many children and teens of different age groups and parents were reached during this timeframe by ‘program x’?	1 item (integer) How many children and teens of different age groups and parents were reached during this timeframe by ‘program x’?	1 item (integer) How many pupils were reached by the ‘program x’ in years 2020/21?	1 item (integer) How many pupils were reached by the programs in years 2020/21?
*Fidelity*				
Adherence: training	3 items (yes/no) Did program administrators receive training by a licensed distributor? Did program administrators receive any kind of training? Did program administrators read the program manual before program start?	3 items (yes/no) Did program administrators receive training by a licensed distributor? Did program administrators receive any kind of training? Did program administrators read the program manual before program start?	3 items (yes/no) Did program administrators receive training by a licensed distributor? Did program administrators receive any kind of training? Did program administrators read the program manual before program start?	Not asked
Adherence: teacher manuals	1 item (yes/no) Were program manuals provided to administrators?	1 item (yes/no) Were program manuals provided to administrators?	1 item (yes/no) Were program manuals provided to administrators?	Not asked
Adherence: participants’ materials	1 item (yes/no) Were materials provided to participants?	1 item (yes/no) Were materials provided to participants?	1 item (yes/no) Were materials provided to participants?	Not asked
Adherence: participant responsiveness	1 item (0–100%) What percentage of participants have attended at least two-thirds of program sessions?	1 item (0–100%) What percentage of participants have attended at least two-thirds of program sessions?	1 item (0–100%) What percentage of participants have attended at least two-thirds of program sessions?	Not asked
Adherence: dosage	2 items (integers) How many sessions does this program have? How many of these sessions were offered to participants?	2 items (integers) How many sessions does this program have? How many of these sessions were offered to participants?	2 items (integers) How many sessions does this program have? How many of these sessions were offered to participants?	Not asked
Program oversight: evaluation	1 item (yes/no) Was the implementation of the program evaluated?	1 item (yes/no) Was the implementation of the program evaluated?	1 item (yes/no) Was the implementation of the program evaluated?	Not asked
Program oversight: quality assurance	1 item (yes/no) Are the results of program evaluation used to make changes to program delivery or teaching practices?	1 item (yes/no) Are the results of program evaluation used to make changes to program delivery or teaching practices?	1 item (yes/no) Are the results of program evaluation used to make changes to program delivery or teaching practices?	Not asked
Program modification	1 item (0–100%) Programs always require some sort of situational adaptation. What percentage of ‘program x’ was adapted?	1 item (0–100%) Programs always require some sort of situational adaptation. What percentage of ‘program x’ was adapted?	1 item (0–100%) Programs always require some sort of situational adaptation. What percentage of ‘program x’ was adapted?	Not asked

#### Adoption

To assess how many EBP were implemented in the communities, respondents were first shown a list of EBP from the evidence registry ‘Grüne Liste Prävention’ (Brender et al., 2024). This registry was developed in 2011 by the Prevention Council of Lower Saxony within the context of CTC. Since 2016, Hannover Medical School has been responsible for the curation of the registry. It contains EBP focused on youth prevention and health promotion. The ‘Grüne Liste Prävention’ is freely accessible and thus could be used by IC as well as CC. In IC, the use of the registry was promoted. School principals received a list of school-wide EBP, teachers were given a list of EBP implemented within a class, and agencies and coalitions were provided with a list of EBP implemented outside of or not primarily within schools. In addition, all respondents were asked if they had implemented any EBP not included on the list. If a school principal or teacher mentioned that an agency or coalition was carrying out an EBP in their school, the relevant agencies and coalitions were specifically asked about this EBP in the survey. At times, during data collection, the same EBP were named multiple times by agents of a community. In these cases, the same prevention programs were only counted once for each community so that overlaps were prevented. Due to the high dropout rate among communities described above, resulting in an unequal number of communities in the two study arms and a slight difference in the average population size between them, we could not directly compare the absolute number of EBP implemented in each study arm, as was done in the CYDS. Instead, we calculated the number of EBP implemented per 10,000 residents for each community. Population size data was obtained from the census records. As an expected outcome, IC are anticipated to implement a higher average number of EBP per 10,000 residents compared to CC.

#### Reach

For each implemented EBP, interviewees were asked about the number of participants reached by the program. Specifically, program agents in coalition and agency interviews reported how many children and teens (by age group) and parents were reached through each program. Principals and teachers were asked about the number of pupils reached by each program. We then calculated the reach for each community by determining how many people per 10,000 residents participated in EBP. On average, IC are expected to reach more people per 10,000 residents with EBP than CC.

#### Fidelity

For each implemented EBP, interviewees were asked about eight aspects of implementation fidelity. If multiple program administrators in the same community carried out the same EBP, their responses to fidelity questions were averaged since it is unlikely that the same EBP was implemented multiple times in a community. (1) Training: An index was used to assess the qualifications of individuals delivering the program. Scores were assigned based on the level of formal training received: a score of 3 was given if the individual was trained by a licensed program provider. If the person was instructed by someone previously trained by a licensed provider, they received a score of 2. A score of 1 was assigned if the individuals had read the program manual before implementation, and a score of 0 was given if none of these criteria were met. The expected outcome is that the IC will have a higher median training score than the CC following the introduction of CTC. (2) Teacher manuals: A higher proportion of program implementers in IC are expected to have received a program manual compared to those in CC. (3) Participant manuals: It is anticipated that a greater proportion of program participants will have received written materials or manuals in IC than in CC. (4) Participant responsiveness: The expected outcome is that a higher proportion of program participants in IC will have attended at least two-thirds of the program sessions compared to CC. (5) Dosage: Typically, in practice, only a small portion of the planned program sessions are completed. For each program, we calculated the percentage of planned sessions that were actually delivered. It is expected that the average dosage percentage will be higher in IC than in CC. (6) Evaluation system: For each EBP, we asked whether the implementation of the program had been evaluated. The expected outcome is that a higher proportion of EBP will undergo evaluation in IC than in CC. (7) Quality assurance: We also asked whether the evaluation results were being used to adapt and improve the program. It is anticipated that a higher proportion of EBP in IC will incorporate these results for quality assurance compared to CC. (8) Program modification: This indicator, not included in the CYDS, tracks the percentage of modifications made to each program. Although we do not have a specific hypothesis on this, we aim to explore whether this indicator shows different trends over time in IC compared to CC. Furthermore, the following two variables were collected for all study communities: (1) Deprivation is measured by the German Index of Socioeconomic Deprivation (GISD) score (Kroll et al., 2017). The GISD score ranges from 0 to 1, with higher values indicating greater deprivation. (2) The number of residents in each community is based on census data.

### Statistical Methods

**Fig. 3:**
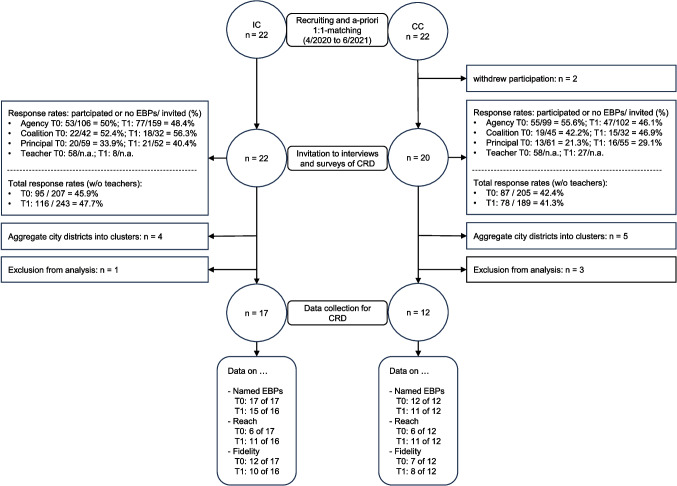
Flow chart of the sampling and data collection process

Statistical analyses were conducted using SPSS Version 29.0 (IBM Corporation, Armonk, NY). The unit of analysis is the community. As described, a number of EBP and reach were standardised by 10,000 residents. Given the lower response rates of interviewees in the CC compared to the IC (see Fig. 3), we analysed the two outcomes after standardising for response rate as well, which was performed by dividing the outcomes by the response rates. Independent samples t-tests were performed to investigate differences between IC and CC at T0 and T1 in the number of EBP and number of participants reached by EBP (Online Resource 1). Paired-sample *t*-tests were used to evaluate any changes from T0 to T1 in the number of EBP and participants reached (Online Resource 2). After regression imputation of missing values (Song & Shepperd, 2007) from communities that lacked data on EBP and their reach for T0 and or T1, the analyses were repeated (Online Resource 3 and Online Resource 4). Differences in implementation fidelity between IC and CC as well as waves were assessed by descriptive analyses. The hierarchical structure of our data as well as sub-group analyses could not be taken into account as our sample size is too small for such analyses.

## Results

Figure 3 illustrates the sampling and data collection process. Initially, the study included 22 matched IC and CC. Due to the COVID-19 pandemic outbreak that occurred in Germany in spring 2020, two CC withdrew from study participation. Consequently, the first wave of data collection on EBP was conducted in 22 IC, but only 20 CC. This sample included four IC and five CC that were districts deriving from five cities. Data collection in these districts was difficult due to various reasons (e.g. program agents did not act in single districts; COVID-19 pandemic). Therefore, for the purpose of analysis, the districts were collapsed back into five large communities, leading to the loss of four IC and five CC. Additionally, in two CC, no data on prevention programs was collected due to poor study participation. One further IC and one CC were excluded from the analysis as the federal state Bavaria dropped out entirely from the study. As a result, we could only include a maximum of 17 IC and 12 CC in the analyses of the baseline data. In addition, two IC and one CC withdrew from the study after the first survey wave, meaning that a maximum of 15 IC and 11 CC could be included in the analyses of this data. As some of the interviewees were unable to provide us with any information on reach and implementation fidelity, the analyses of these two outcomes are based on fewer communities.

Figure 3 also depicts the number of study participants of each survey type (i.e. agency, coalition, principal, and teacher interview) with the corresponding response rates. As teachers were invited by the principals, no response rate was calculated. Thus, the overall response rates do not include the number of teachers. In total, the response rate was higher in IC (T0 = 39%; T1 = 34.6%) than in CC (T0 = 30%, T1 = 33.3%) for the first and second survey waves. Interestingly, response rates decreased from T0 to T1 in all but principal interviews.

The community samples for T0 and T1 are described in Table 2. Based on descriptive statistics, deprivation appears a little bit higher mostly in the IC samples and CC samples had on average a little bit larger population sizes.

**Table 2:** Community sample description

	Communities with data on …					
	No. of implemented EBP		No. of reached persons		Implementation fidelity	
	IC	CC	IC	CC	IC	CC
	T0 (wave 1)					
	*n* = 17	*n* = 12	*n* = 6	*n* = 6	*n* = 12	*n* = 7
Population size (median)	11,977	13,884	9932	13,884	12,183.5	13,972
Deprivation (mean)	0.5087	0.4325	0.6029	0.4701	0.5128	0.3876
Federal states (*n*)						
Baden Wurttemberg	7	5	3	2	7	4
Lower Saxony	9	6	3	4	4	3
Rhineland-Palatinate	1	1	0	0	1	0
	T1 (wave 2)					
	*n* = 15	*n* = 11	*n* = 11	*n* = 11	*n* = 10	*n* = 8
Population size (median)	11,977	13,796	11,977	13,796	12,183,5	13,884
Deprivation (mean)	0.4922	0.4977	0.4829	0.4633	0.4679	0.4700
Federal states (*n*)						
Baden Wurttemberg	6	2	6	2	6	2
Lower Saxony	8	8	4	8	3	6
Rhineland-palatinate	1	1	1	1	1	0

**Fig. 4:**
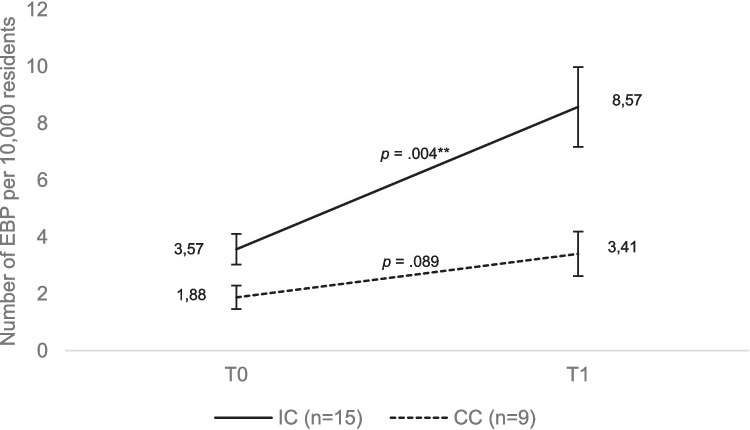
Means with standard errors of the numbers of implemented EBP per 10,000 residents in IC and CC at wave 1 (T0) and wave 2 (T1)

A significant increase from T0 to T1 in the number of EBP per 10,000 residents was shown in IC, *t*(14) =  − 3.472, *p* = 0.004, but not in CC, *p* = 0.089 (Fig. 4). This result remains robust, even when data are standardised with regard to response rates (Online Resource 2). After data imputation (Online Resource 4), the increase becomes statistically significant in CC as well (*p* = 0.033). When analysing differences between IC and CC and including communities for which no longitudinal data is available, the IC and CC at T1 do not differ significantly from each other (Online Resource 1).

**Fig. 5:**
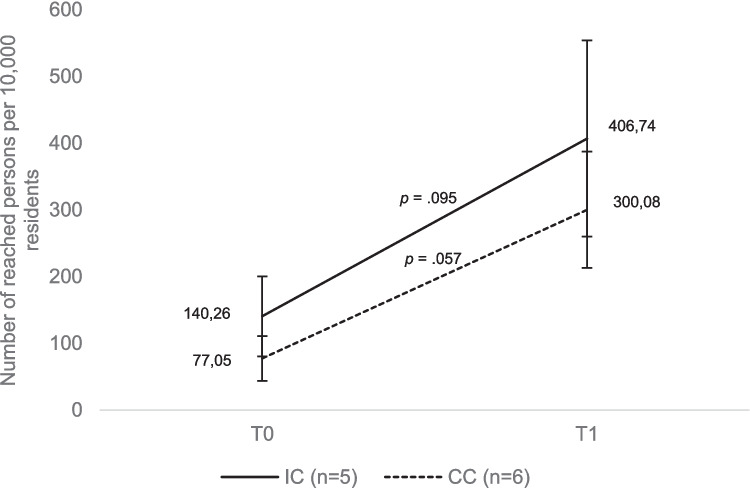
Means with standard errors of the reached persons per 10,000 residents in IC and CC at wave 1 (T0) and wave 2 (T1)

Neither in IC (*p* = 0.095) nor in CC (*p* = 0.057) significant changes in the number of persons reached per 10,000 residents were shown (Fig. 5). This result also remains robust when data are standardised with regard to response rates (Online Resource 2). After data imputation (Online Resource 4), the increase becomes statistically significant in CC as well (*p* < 0.001). Again, when analysing differences between IC and CC and including communities for which no longitudinal data is available, the IC and CC do not differ significantly from each other at T1 (see Online Resource 1).

Table 3 shows that the outcomes for implementation fidelity hardly differ between IC and CC as well as between T0 and T1 and, above all, do not differ systematically. At both waves, in IC as well as CC, a majority of staff were trained by a licensed distributor as indicated by the medians of 3. At T0, IC appear to have a somewhat higher quality of training. As for materials, the provision of teacher manuals and participant materials declined in IC but increased in CC. Participant responsiveness declined in both IC and CC, but more strongly in CC. Dosage appeared to increase minimally in IC but declined in CC from wave 1 to wave 2. Overall adherence rates appear high in IC as well as CC. Percentages of program evaluation increased in both IC and CC, but in CC the increase appears much more strongly (+ 48.81%). On the other hand, quality assurance declined in both study arms but is overall still quite high. Program modification rates declined in IC as well as CC. In IC, however, this drop was more noticeable.

**Table 3:** Fidelity outcomes in IC and CC at T0 and T1

	T0 (wave 1)		T1 (wave 2)	
Outcomes	IC	CC	IC	CC
*Adherence*				
Training^a^	3 (2.5–3.0) (*n* = 12)	3 (2.17–3.0) (*n* = 7)	3 (2.0–3.0) (*n* = 10)	3 (2.0–3.0) (*n* = 8)
Teacher manuals^b^	100 ± 0 (*n* = 12)	80.95 ± 37.79 (*n* = 7)	83.3 ± 35.35 (*n* = 9)	100 ± 0 (*n* = 6)
Participant materials^b^	100 ± 0 (*n* = 12)	96.43 ± 9.45 (*n* = 7)	98.57 ± 4.52 (*n* = 10)	100 ± 0 (*n* = 8)
Participant responsiveness^b^	94.16 ± 8.48 (*n* = 12)	86.84 ± 10.07 (*n* = 5)	91.63 ± 3.54 (*n* = 8)	79.6 ± 37.46 (*n* = 5)
Dosage^b^	97.05 ± 6.78 (*n* = 11)	90.06 ± 13.01 (*n* = 6)	99.38 ± 1.76 (*n* = 8)	81.25 ± 14.23 (*n* = 4)
*Program oversight*				
Evaluation system^b^	82.5 ± 33.44 (*n* = 10)	42.86 ± 53.45 (*n* = 7)	88.88 ± 22.05 (*n* = 9)	91.67 ± 20.41 (*n* = 6)
Quality assurance^b^	100 ± 0 (*n* = 8)	100 ± 0 (*n* = 3)	88.88 ± 33.33 (*n* = 9)	90 ± 22.36 (*n* = 5)
*Program modification*^b^	34.92 ± 22.49 (*n* = 10)	28.64 ± 14.92 (*n* = 6)	16.17 ± 27.67 (*n* = 10)	23.13 ± 17.97 (*n* = 8)

^a^level of measurement: ordinal (0 = no training, 1 = program manual read, 2 = trained by distributor, 3 = trained by licensed distributor), descriptive statistics: median and range

^b^level of measurement: metric (0–100), descriptive statistics: average percentage and standard deviation

## Discussion

In our study, we conducted the first analysis in Germany to assess whether introducing the CTC approach impacts communities in terms of the adoption, reach and implementation fidelity of EBP. When interpreting our results it should be considered that the COVID-19 pandemic slowed down the introduction of CTC considerably. Our process evaluation shows that only four IC had reached CTC-phase 5 (implementation of EBP) by the beginning of 2024 (Ünlü et al., 2024). Of these, two IC had already reached CTC-phase 5 during our baseline measurements.

In our study communities, where longitudinal data on the adoption and reach of EBP were collected, the following findings emerged: In the 2020/21 school year, the average number of EBP implemented per 10,000 residents was 3.57 in IC and 1.88 in CC. By the 2022/23 school year, there was a statistically significant increase to 8.57 (*p* = 0.004) in IC and a non-significant increase to 3.41 (*p* = 0.089) in CC. IC differed statistically significantly from CC at T1 with regard to the number of EBP implemented. We repeated these analyses after regression-analytically estimating the number of EBP implemented in those communities for which we were unable to collect data on EBP at T0 and/or T1 (Online Resource 4). Even though the increase in EBP in CC after imputation becomes statistically significant, the increase in IC is twice as high. This suggests an association between the introduction of the CTC approach in communities and the community-wide adoption of EBP. In the CYDS, IC had implemented 17 EBP at T0 (2002) and 36 at T1 (2005), while CC had implemented 11 EBP at T0 and 24 at T1 (Fagan et al., 2011). The differences between IC and CC in the number of EBP implemented were not statistically significant at either time point. These differences in the CYDS were only statistically significant at T2 (2007) when IC had implemented 44 EBP and CC 19 EBP. This means that in the CYDS, as in our study, a general trend was found from T0 to T1 with regard to an increasing spread of EBP in communities. While the CYDS did not find a higher spread of EBP in IC compared to CC three years after the start of CTC implementation, our study shows a higher spread of EBP in IC compared to CC just two years after the start of CTC implementation. This could be due to the fact that our intervention group also includes communities that were in CTC-phases one (‘get started’), two (‘get organised’), three (‘develop a profile’) or four (‘create a plan’) before T0. In addition, we cannot completely rule out confounding due to our study design. Data not analysed here, which we collected as part of our study, shows that due to the COVID-19 pandemic in 2020, some preventive measures were initially suspended in both IC and CC (Online Resource 5). In the two subsequent years, preventive measures were then expanded in IC and CC. We therefore assume that the pandemic had no differentiating effects on IC and CC that could have distorted results.

In terms of reach, the average number of people reached per 10,000 residents in the 2020/21 school year was 140 in IC and 77 in CC. By the 2022/23 school year, a non-significant increase to 407 (*p* = 0.095) was observed in IC and a non-significant increase to 300 (*p* = 0.057) in CC. Once again, we repeated these analyses after regression-analytically estimating the number of people reached with EBP for those communities for which we were unable to collect data on the number of people reached with EBP at T0 and/or T1 (Online Resource 4). After this imputation of missing values, the increase in the number of people reached with EBP becomes statistically significant in both IC and CC. The increase is slightly higher in IC than in CC, although IC and CC are not statistically different at T1 either. In the CYDS, the IC reached 3,454 people with EBP at T0 and 5,522 at T1, and the CC reached 3,333 at T0 and 6,084 at T1 (Fagan et al., 2011). At neither time point were these differences between IC and CC statistically significant. At T2, 11,261 people were reached with EBP in IC and 3,864 in CC. At this time point, this difference was statistically significant. Our finding that IC do not reach significantly more people with EBP than CC two years after the start of CTC implementation is consistent with the results of the CYDS. We should therefore continue our study and examine whether, as in the CYDS, more people with EBP are reached in IC than in CC five years after the start of CTC implementation. Since we have already observed a greater spread of EBP in IC compared to CC two years after the start of the implementation of CTC, it needs to be explained why more people are not already being reached with EBP in IC than in CC. This may be due to the fact that we were unable to collect data on this question for many communities, resulting in a high random error or uncontrollable confounding. We are unable to clarify this at the moment. It is also possible that due to the COVID-19 pandemic, which persisted in Germany into the 2022/2023 school year, EBP reached fewer people than it would have been the case without the pandemic. This could be clarified by conducting a third survey wave in the future.

Since most interview respondents did not provide information on implementation fidelity for many EBP, we had too few data points to conduct inferential statistical analyses. Our purely descriptive analyses on implementation fidelity show no indication that IC and CC differed from each other or had different time trends. In both study arms, implementation fidelity was relatively high at both survey time points. Similarly, the CYDS (Fagan et al., 2011) found no systematic effects of CTC on implementation fidelity. In this regard, our findings are also consistent with those of the CYDS.

The main limitations of our study are the following. Our study does not rely on randomised allocation to the two study arms, so it remains possible that the stronger improvements in IC compared to CC are not caused by CTC, but by unobserved confounders. Since our original 1:1 matching could not be maintained due to high non-response and loss-to-follow-up (i.e. loss of communities from start of the study to T1), between-group comparisons became less reliable, and the observed effects may reflect underlying differences caused by selection and/or attrition bias rather than caused by CTC. It might be postulated that CC and respondents with less interest in EBP and implementation fidelity were less likely to participate in our study. If they had participated, these communities would have likely reported no implementation of EBP, which could have enhanced the observed differences between IC and CC in favour of IC and led to an underestimation of the true effect of CTC. However, if lower-performing IC or less-engaged CC disproportionately dropped out, it could lead to an inflation of observed differences, i.e. an overestimation of CTC. We, therefore, performed regression imputation for missing data of EBP and reach, followed by a repetition of previous analyses (Online Resources 3 and 4). This shows that our results remain stable after imputation of missing values. Lastly, it should also be noted that our study is not based on a representative sample of German communities, so our results can be generalised only to a limited extent.

Our study has several strengths. It draws on a highly heterogeneous sample of German communities from three different federal states. For data collection, we used methods and instruments that had already been validated in the CYDS. Additionally, it is the first study in Germany to examine the effects of CTC on the adoption and reach of EBP, as well as on the fidelity of EBP implementation.

## Conclusion

In our study of CTC’s early effects, we found indications that CTC promotes the adoption of EBP. However, we did not find indications that CTC promotes the reach of EBP and the fidelity of EBP implementation. To gain a clearer understanding of CTC’s impact on EBP adoption, a follow-up is needed.

## Supplementary Information

Below is the link to the electronic supplementary material.

Supplementary file1 (PDF 189 KB)

Supplementary file2 (PDF 187 KB)

Supplementary file3 (PDF 190 KB)

Supplementary file4 (PDF 187 KB)

Supplementary file5 (PDF 292 KB)

## Data Availability

The datasets analysed during the current study are available upon reasonbale request.

## Declarations

### Clinical Trial Registration

German Clinical Trials Register: DRKS00022819, 2022–03-08.
