# Supplementary material for: Early Effects of Communities That Care on the Adoption and Implementation Fidelity of Evidence-Based Prevention Programs in Communities: Results from a Quasi-experimental Study
Source: Prev Sci. 2025 Jul 1;26(6):873–85. doi: 10.1007/s11121-025-01823-w (PMC12394388; doi:10.1007/s11121-025-01823-w)
Supplement: Supplementary file 2 — Supplementary file2 (PDF 187 KB) [file 11121_2025_1823_MOESM2_ESM.pdf]

Supplementary Material 2

Article Title: Early Effects of Communities That Care on the Adoption and Implementation Fidelity of Evidence-Based Prevention Programs in Communities. Results from a Quasi-Experimental Study

Journal: Prevention Science

Authors: Decker, L., von Holt, I., Ünlü, S., Walter, U., Röding, D.

Affiliation: Hannover Medical School

Mail: decker.lea@mh-hannover.de

Online Resource 2 Changes from T0 to T1 in the number of adopted EBP and reached persons in IC and CC (unstandardised vs. standardised)

	IC		CC	
	T0	T1	T0	T1
Outcomes	Mean (SD)	Mean (SD)	Mean (SD)	Mean (SD)
Adoption: No. of EBP per 10,000 residents				
unstandardised	3.57 (2.16)	8.57 (5.63)	1.87 (1.32)	3.41 (2.49)
	t(14) = -3.472, p = .004, n = 15		*t(8) = -1.936, p = .089, n = 9*	
standardised	7.76 (4.69)	17.97 (11.79)	4.43 (3.1)	8.25 (6.03)
	t(14) = -3.366, p = .005, n = 15		*t(8) = -2.003, p = .080, n = 9*	
Reach: No. of reached persons per 10,000 residents				
unstandardised	140.26 (149.36)	406.74 (367.83)	77.04 (90.48)	300.07 (234.32)
	t(4) = -2.179, p = .095, n = 5		*t(5) = -2.469, p = .057, n = 6*	
standardised	305.58 (325.4)	852.71 (771.14)	181.71 (213.39)	726.57 (567.36)
	t(4) = -2.156, p = .097, n = 5		*t(5) = -2.492, p = .055, n = 6*	
