# Supplementary material for: Early Effects of Communities That Care on the Adoption and Implementation Fidelity of Evidence-Based Prevention Programs in Communities: Results from a Quasi-experimental Study
Source: Prev Sci. 2025 Jul 1;26(6):873–85. doi: 10.1007/s11121-025-01823-w (PMC12394388; doi:10.1007/s11121-025-01823-w)
Supplement: Supplementary file 3 — Supplementary file3 (PDF 190 KB) [file 11121_2025_1823_MOESM3_ESM.pdf]

Supplementary Material 3

Article Title: Early Effects of Communities That Care on the Adoption and Implementation Fidelity of Evidence-Based Prevention Programs in Communities. Results from a Quasi-Experimental Study

Journal: Prevention Science

Authors: Decker, L., von Holt, I., Ünlü, S., Walter, U., Röding, D.

Affiliation: Hannover Medical School

Mail: decker.lea@mh-hannover.de

Online Resource 3 Comparison of IC and CC regarding the adoption and reach of EBP at T0 and T1 (unstandardised vs. unstandardised and imputated)

	T0 (Wave 1)		T1 (Wave 2)	
Outcomes	IC<br>Mean (SD)	CC<br>Mean (SD)	IC<br>Mean (SD)	CC<br>Mean (SD)
Adoption: No. of EBPs per 10,000 residents				
unstandardised	3.59 (2.43)<br>n =17	1.77 (1.23)<br>n =12	8.57 (5.63)<br>n =15	4.83 (5.07)<br>n =11
	t(24.941) = 2.654, p = .014		*t(24) = 1.745, p = .094*	
unstandardised (imputated)	3.59 (2.43)<br>n =17	1.91 (1.24)<br>n =14	8.59 (5.32)<br>n =17	4.47 (4.51)<br>n =14
	t(24.681) = 2.492, p = .020		*t(29) = 2.293, p = .029*	
Reach: No. of reached persons per 10,000 residents				
unstandardised	180.36 (165.81)<br>n = 6	77.04 (90.48)<br>n = 6	273.4 (310.28)<br>n = 11	258.02 (241.08)<br>n = 11
	t(7.735) = 1.34, p = .218		*t(20) = 0.13, p = .898*	
unstandardised (imputated)	82.96 (124.24)<br>n = 17	55.69 (68.68)<br>n = 14	308.16 (271.5)<br>n = 17	266.27 (212.08)<br>n = 14
	t(29) = .733, p = .469		*t(29) = .471, p = .641*	
