# Supplementary material for: Early Effects of Communities That Care on the Adoption and Implementation Fidelity of Evidence-Based Prevention Programs in Communities: Results from a Quasi-experimental Study
Source: Prev Sci. 2025 Jul 1;26(6):873–85. doi: 10.1007/s11121-025-01823-w (PMC12394388; doi:10.1007/s11121-025-01823-w)

Supplementary Material 5

Article Title: Early Effects of Communities That Care on the Adoption and Implementation Fidelity of Evidence-Based Prevention Programs in Communities. Results from a Quasi-Experimental Study

Journal: Prevention Science

Authors: Decker, L., von Holt, I., Ünlü, S., Walter, U., Röding, D.

Affiliation: Hannover Medical School

Mail: decker.lea@mh-hannover.de

Online Resource 5

This figure depicts changes in the extent of prevention and health measures between years in IC and CC. These results are based on the instrument of the Community Key Informant Interview (CKI). At T0, interviewees were asked to respond to the following items: '(1) When comparing today's situation with the year 2019, did the extent of prevention and health promotion measures for youth in your community change?; (2) When comparing today's situation with the year 2020, did the extent of prevention and health promotion measures for youth in your community change?'. At T1, interviewees were asked 'When comparing today's situation with the last year (2022), did the extent of prevention and health promotion measures for youth in your community change?'. If interviewees responded 'yes', they were asked a follow-up question: 'How did the extent change? Would you say it (1) drastically increased (2) somewhat increased (3) somewhat decreased (4) drastically decreased'. The results of the follow-up question are depicted in this figure. The following description of results for changes from year 2019 to 2021 exemplifies how the figure should be understood. The majority of interviewees (87.4%) indicated changes in measures from 2019 to 2021. Twenty-six percent of IC and 26.7% of CC indicated a decrease in prevention and health measures for youth, whereas only 17.4% of IC and 17.3% of CC indicated an increase in measures.

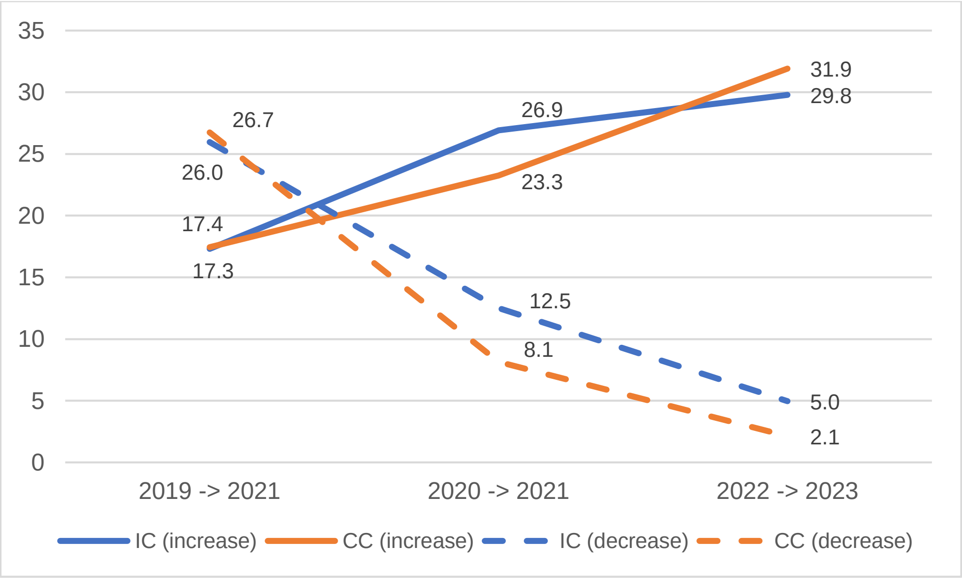

Supplement: Supplementary file 5 — Supplementary file5 (PDF 292 KB) [file 11121_2025_1823_MOESM5_ESM.pdf]
